# Rural and regional maternity managers’ and educators’ views of the Maternity and Newborn Emergencies (MANE) education program in Victoria, Australia: a qualitative descriptive study

**DOI:** 10.1186/s12913-023-10466-y

**Published:** 2024-01-03

**Authors:** Stefanie A Zugna, Helen L McLachlan, Meabh Cullinane, Michelle Newton, Della A Forster

**Affiliations:** 1 Judith Lumley Centre, School of Nursing and Midwifery, La Trobe University, Bundoora, VIC 3086 Australia; 2 Royal Women’s Hospital, Parkville, VIC Australia

**Keywords:** Interprofessional clinical education, Multidisciplinary education, Teamwork, Maternity emergencies, Neonatal emergencies, Clinical governance, Safety culture, Management

## Abstract

**Background:**

Australia has one of the lowest perinatal morbidity and mortality rates in the world, however a cluster of perinatal deaths at a regional health service in the state of Victoria in 2015 led to state-wide reforms, including the introduction of the Maternity and Newborn Emergencies (MANE) program. MANE was a 2-day interprofessional maternity education program delivered by external expert facilitators to rural and regional Victorian maternity service providers. An independent evaluation found that the MANE program improved the confidence and knowledge of clinicians in managing obstetric emergencies and resulted in changes to clinical practice. While there is a large volume of evidence that supports the use of interprofessional education in improving clinicians’ clinical practice, the impact of these programs on the overall safety culture of a health service has been less studied. Managers and educators have an important role in promoting the safety culture and clinical governance of the heath service. The aim of this study, therefore, was to explore Victorian rural and regional maternity managers’ and educators’ views and experiences of the MANE program.

**Methods:**

Maternity managers and educators from the 17 regional and rural health services across Victoria that received the MANE program during 2018 and 2019 were invited to participate. Semi-structured interviews using mostly open-ended questions (and with a small number of fixed response questions) were undertaken. Qualitative data were transcribed verbatim and analysed thematically. Descriptive statistics were used for quantitative data.

**Results:**

Twenty-one maternity managers and educators from the 17 health services participated in the interviews. Overall, participants viewed the MANE program positively. Four themes were identified: the value of external facilitation in providing obstetric emergency training; improved awareness and understanding of clinical governance; improved clinical practice; and the importance of maintaining the program. Participants agreed that MANE had improved the confidence (94%) and skills (94%) of clinicians in managing obstetric emergencies, as well as confidence to escalate concerns (94%), and most agreed that it had improved clinical practice (70%) and teamwork among attendees (82%).

**Conclusion:**

Maternity managers and educators were positive about MANE; they considered that it contributed to improving factors that impact the safety culture of health services, with delivery by external experts considered to be particularly important. Given the crucial role of maternity managers and educators on safety culture in health services, as well in program facilitation, these findings are important for future planning of maternity education programs across the state.

**Trial registration:**

Trial registration was not required for this study.

## Background

The effectiveness of multidisciplinary obstetric emergency education programs on improving clinicians’ knowledge and confidence has been studied extensively [1]. A systematic review assessed the effectiveness of obstetric emergency training, reporting participants’ reactions to training was positive, led to increased knowledge and skills and improved clinical practice [1]. One obstetric emergency training program implemented widely across the state of Victoria, Australia is the Practical Obstetric Multi-Professional Training (PROMPT) program. Clinicians attend this program within their health service, with training conducted by educators within the service, utilising a ‘train the trainer’ model. An evaluation of PROMPT in a Victorian maternity service found that participation resulted in improvement in clinical and non-technical skills, although there was no significant change in clinical outcomes [2]. An earlier evaluation investigated the impact of PROMPT in eight Victorian metropolitan and regional hospitals on organisational culture and perinatal outcomes and found improvements in organisational culture measured using the Safety Attitudes Questionnaire (SAQ), as well as improvements in neonatal outcomes [3].

While there is a large volume of evidence to support the role that obstetric emergency training programs play in improving clinicians’ clinical practice, the impact of these programs on the overall safety culture of a health service has been less studied. Safety culture is defined as ‘a product of individual and group values, attitudes, perception, competencies and patterns of behaviour that determine the commitment to, and the style and proficiency of an organisation’s health and safety management’ [4]. A recent study used a pre-post assessment of culture (Hospital Survey on Patient Safety Culture) to evaluate the effectiveness of an education program called Team Strategies and Tools to Enhance Performance and Patient Safety (TeamSTEPPS) on the safety culture in two Swiss maternity wards [5]. It found the program led to significant improvements in patient safety culture in the ward receiving the intervention compared to the control ward [5].

Although there is some evidence that education programs can influence safety culture, a recent cross-sectional secondary analysis of nurses’ views on patient safety culture concluded that managers have the strongest influence on the overall safety culture in a health service [6]. A qualitative study of nurse managers found that managers believed they had a significant impact on the safety culture with their unit [7]. The safety culture of a health service is inextricably linked to the clinical governance in that health service. Organisations with strong clinical governance structures generally have a good safety culture [8]. The Victorian Clinical Governance Framework (VCGF) describes clinical managers as having a crucial role in creating a culture of safety, transparency, accountability, teamwork and collaboration [9].

## The Maternity and Newborn Emergencies (MANE) program

In Victoria, Australia around 20% of women birth in regional and rural health care services [10]. Victoria’s perinatal mortality rate which includes stillbirths (i.e., a fetal death prior to 20 or more completed weeks of gestation or of 400 g or more birthweight [11]) and deaths of live-born babies within the first 28 days of life, is among the lowest in Australia (8.9 per 1000 births in 2020) and comparable with other countries of similar socioeconomic status [12]. A cluster of perinatal (stillbirth or early neonatal) deaths at one of the state’s regional health services in 2015, however, highlighted a number of areas of concern, including inadequate midwifery education and inadequate clinical governance frameworks [13]. Following investigation, broader reforms in the state’s public hospital governance system resulted, including the establishment of Safer Care Victoria (SCV), the state’s lead agency for monitoring and improving quality and safety in health care (described in detail in a discussion paper by the authors of this manuscript [14]). As well as these reforms, targeted initiatives were introduced to address concerns specific to maternity services. One of these initiatives was the implementation the Maternity and Newborn Emergencies (MANE) program.

The MANE program aimed to improve the safety culture of maternity services by providing high quality maternity and neonatal emergency training, as well as educating clinicians on recognition and response to clinical deterioration and improving their understanding of and engagement with clinical governance and risk management principals. A detailed description of the MANE program has been published previously [15].

The Victorian Department of Health (formerly known as the Department of Health and Human Services) mandated that MANE be delivered to all low to medium risk maternity service providers located in regional and rural Victoria services. The program was facilitated by the Maternity Services Education Program (MSEP) team, a Victorian Department of Health funded initiative run by the Royal Women’s Hospital, Melbourne [15]. Midwives and obstetricians from MSEP facilitated the maternity emergencies component of the program, whilst neonatologists and neonatal nurses from the Paediatric Infant Perinatal Emergency Retrieval (PIPER) service based at the Royal Children’s Hospital delivered the neonatal component of MANE.

The MANE program was open to any clinician caring for maternity and neonatal patients including midwives, nurses, obstetric trainees, obstetricians, paediatricians, general practitioners, paramedics, and students. The program, run over two days, consisted of presentations, workstations, and simulations, with both core components and elective modules chosen by the health service. In keeping with the broader aim of MANE to improve the safety of maternity services, on the last day of the program attendees were given the opportunity to highlight clinical and governance concerns to facilitators during a feedback session facilitated by MSEP utilising a ‘Review and Response Tool’. This tool gave attendees the opportunity to identify strengths and issues in their health service as categorised by each of SCV’s five domains of good clinical governance: consumer participation; clinical practice; risk management; workforce; and leadership and culture [9]. The data obtained from this session were de-identified, collated and fed back by MSEP to the executive and management staff within the service.

An evaluation of the MANE program was conducted by the authors of this paper using the Kirkpatrick Evaluation Model to determine the effectiveness of MANE in relation to: governance changes at the health service; organisational behaviour change; clinician behaviour change; multidisciplinary education, teamwork and collaboration across teams and disciplines; individual clinician education and practical use of skills; and consumer experience and satisfaction with quality of care [15]. A study protocol paper published in 2020 described how the Kirkpatrick Evaluation Model was to be used to address the aims of the evaluation, and outlined the components of data collection [15]. Data were collected from 17 maternity services that received the MANE program during 2018 and 2019. One aspect of the evaluation included clinicians’ self-reported confidence and knowledge in managing obstetric emergencies using surveys prior to, and at three time points after MANE (immediately post-MANE, 6-months post-MANE and 12-months post-MANE). Only clinicians who attended the MANE program completed the pre-MANE and post-MANE surveys, and whilst all maternity and newborn care clinicians were invited to complete the 6 and 12-month post-MANE survey, only data collected from MANE attendees were reported. Findings from these surveys were reported in Cullinane et al. [16]. Response rates varied, with high response rates for the pre-MANE and post-MANE surveys (84% [294/350] and 81% [282/530] respectively) compared to 22% and 21% for the 6 and 12-month post-MANE surveys. There were both immediate and sustained changes in terms of confidence and knowledge of obstetric and newborn emergencies resulting from MANE. For example, pre-MANE, less than 10% of participants assessed their knowledge of newborn resuscitation as excellent, compared to more than 31% post-MANE, 20% 6-months post, and 30% 12-months post-MANE. Likewise, around 45% of participants felt confident managing newborn resuscitation pre-MANE, compared to more than 80% at all three timepoints post-MANE [16]. The surveys also measured the safety climate of the service using the SAQ [17] which showed a large variation in safety climates across the health services, with some health services having strong safety climates, and others weaker. It must be noted, however, that the low response rate at the 6 and 12-month post-MANE surveys, and other independent factors such as other education and quality improvement programs being offered in some sites were limitations to the study.

While the clinician survey data component of the evaluation of the MANE program found that MANE improved the confidence and knowledge of *clinicians* in managing obstetric emergencies, this paper reports on another aspect of the evaluation, in which *managers’ and educators’* views and experiences of the MANE program at their health services were explored. This is important to explore given the crucial role that managers and educators play in safety culture, clinical governance and program facilitation.

## Methods

### Aim

To describe Victorian rural and regional maternity managers’ and educators’ views of the Maternity and Newborn Emergencies (MANE) education program at their health service.

### Study design

A qualitative descriptive study using semi-structured telephone interviews with the inclusion of fixed response statements.

### Setting

The study was conducted at low to medium risk maternity services across rural and regional Victoria, Australia.

### Sampling and recruitment

#### Participants

Eighteen health services received MANE in 2018 and 2019. One service was excluded from the evaluation as there were other interventions introduced at around the same time to improve safety in that service. Maternity managers and educators at the remaining 17 services were eligible to participate.

#### Recruitment

The research team informed the eligible health services about the evaluation via a letter to the service’s Chief Executive Officer, with the option for services to opt out if they did not wish to participate. If no ‘opt out’ was received, the maternity manager was invited via email to participate in the study. The managers then identified who would be the most appropriate person to participate, according to who had the most knowledge of the MANE program and its impact on their health service. The identified staff were emailed by the project coordinator around three months after MANE had been delivered at their health service to arrange a suitable time for a telephone interview, which was planned to take place approximately four months after the MANE program was conducted at their service. At the commencement of the telephone interview, participants were informed more about the background for the study. Given this study was deemed low-risk research, verbal informed consent was obtained before the interview, and permission sought to audio record for transcription purposes of the data as approved by the La Trobe University Science, Health and Engineering College Human Ethics Sub-Committee.

### Data collection tools and processes

Semi-structured interviews were conducted by telephone. The interviews consisted of mostly open-ended questions with a small number of fixed-response statements.

The interview guide was developed specifically for the study and the open-ended questions explored participant’s views and experiences on the organisation’s decision to participate in MANE, expectations of MANE, perceived changes around clinical governance and organisational change as a result of the MANE program, the role of PROMPT and MANE (given most maternity services across Victoria also run the PROMPT program), and sustainability of MANE for the future. Additionally, five statements using a five-point Likert-type scale measured participants’ perception of changes in clinicians’ confidence and skill acquisition, and changes to teamwork and collaboration resulting from MANE. They were asked to rate their responses from ‘Strongly agree’ to ‘Strongly disagree’. Some interviews included more than one participant at the health service. In interviews where there was more than one participant, participants conferred to make sure they were in agreeance on the rating representing their combined views.

### Data analysis

Interviews were recorded and transcribed verbatim, and transcripts were checked for accuracy by two members of the research team. Potentially identifying information about individuals and individual hospitals were removed, and each health service identified only by number within the evaluation (health service 1 to health service 17). Interview data were then analysed independently by two members of the research team.

Data were analysed using thematic analysis [18]. After familiarisation with the data (by reading and re-reading the transcripts), responses were initially coded by question, the codes grouped into categories, and then data combined to obtain themes using inductive thematic analysis [19]. Themes were then reviewed and cross-checked between the two researchers to check for consistency before they were finalised. Direct quotes have been used to illustrate the themes identified, with the position of the interviewee and the de-identified health service number used as an identifier. The Likert-type scale statements were analysed using simple descriptive statistics.

The researchers analysed interview data independently, then corroborated their findings to ensure confirmability of the results. The use of a different researcher in the data collection and data analysis steps has ensured investigator triangulation, strengthening the credibility of the findings [20].

## Results

### Participants

The interviews were conducted from July 2018 to May 2020 by one or two members of the research team. All of the 17 health services that were approached agreed to participate.

Ten interviews were conducted with the maternity manager only, two were with a maternity manager and maternity educator present, four with a midwifery educator only, and one was conducted with the maternity manager, midwifery educator and a member of the executive team, totalling 21 participants representing 17 health services. On average, interviews were 22 min in length (range 12 to 36 min).

### Themes

Analysis of the interview data identified four themes: *the value of external facilitation in providing obstetric emergency training; improved awareness and understanding of clinical governance; improved clinical practice;* and *the importance of maintaining the program.*

#### The value of external facilitation in providing obstetric emergency training

Participants considered that MANE provided quality education. They highly valued the expertise brought by the MSEP and PIPER facilitators i.e., the benefit of ‘external eyes’ coming into their service.

We participated in MANE primarily for the ongoing education and upskilling of our staff … I think it’s also very important to have an external … set of eyes and ears and … facilitators from time to time. (Maternity manager, health service 4)

We don’t have a lot of education up here which is focused on midwifery emergencies and neonatal resuscitation. Often, we have to go elsewhere to attend so it’s very good that [MSEP] comes up. (Midwifery educator, health service 16)

Participants considered that a key role of MANE was to provide maternity and neonatal emergency scenario simulation/education that was tailored to rural health services with the aim of improving the skills of clinicians. Again, participants identified the important role of MANE in providing an *external* view of the maternity unit of their health service, as well as an opportunity for services to reflect on their internal processes.

It’s to provide … obstetric emergency education to smaller less resourced hospitals. And the intention is to, to bring that expertise from the larger places to provide that education to the smaller rural places that don’t have the same sort of resources or … education … I guess the ultimate aim is to assist us in providing safer care to our patients. (Maternity manager, health service 13)

Overwhelmingly, participants indicated that MANE either met or exceeded expectations.

My expectations were really on the education around the emergency response and simulation, so to get the bonus … about looking at clinical governance and the opportunity for reflection at the end, that made it a whole package for the day, it wasn’t just ‘let’s do the simulations and go’. So, I think that was above my expectations I’d have to say. (Maternity manager, health service 4)

The MANE program was also perceived as a good opportunity for smaller services to build links and network with bigger centres.

I think it’s a great program … it’s a link to that outside world of maternity and it makes you feel connected to the bigger centres, and it makes you feel not forgotten, and it would be really sad to not have that I think. (Maternity manager, health service 1)

#### Improved awareness and understanding of clinical governance

A key theme from the interviews was that participants considered that MANE had positively impacted on attendees’ awareness and understanding of clinical governance. This included increased involvement and engagement in clinical governance activities as well an increased awareness of the importance of monitoring clinical outcome data.

I’m not sure … 100% whether everybody understands what clinical governance is, but more and more people are, and I think MANE is just another avenue … it does make people more aware of it and I think it definitely has done that for us. (Maternity manager, health service 1)

Absolutely [MANE had an impact on clinician’s understanding of their role in clinical governance]. I think there is more awareness of statistics. (Midwifery educator, health service 12)

[MANE] certainly has impacted on the [number] of midwives that have perhaps wanted to be involved in [Perinatal Audit Meetings] you know, have engaged over the last … meeting and the meeting before. (Maternity manager, health service 8)

#### Improved clinical practice

Responses to the closed-ended questions demonstrated that all participants agreed that MANE improved the skills and confidence of clinicians. This is supported in the responses to the open-ended questions with a number of participants commenting that this was important due to small numbers of births at their services, but also that it was challenging to sustain the confidence, and that the program should be undertaken regularly. That is, there was a view from some that although there was an improvement in skills and confidence, these were potentially only short term due to the lack of exposure of these scenarios in the smaller health services.

In places like this that only have … 90 to 100 births a year … it’s hard to sustain that level of confidence and level of skill. (Midwifery educator, Health service 16)

I find that it actually raises everyone’s confidence level initially, but it’s something that we need to do each year to keep that confidence up. (Midwifery educator, Health service 7)

The Review and Response Tool, which aimed to give clinicians the opportunity to identify strengths and issues in their health service, was completed on the second day of each MANE workshop. Most participants considered that MANE had resulted in changes to clinical practice in their unit. These included improvements to clinical policies and procedures; improvement of equipment/ room layout; increase in midwives working to their full scope of practice; increased consumer involvement with the service; and the introduction of further education in the service.

We have purchased a syringe driver for the department … we’ve secured … two clocks in each labour ward. We’ve revised the emergency trolley and the location … I think I think the most important one having those … documentation tools readily available in the labour ward. (Maternity manager, health service 2)

We have purchased a [new] machine … So that’s definitely one thing that we have implemented from MANE … with their support as well it was a quicker process than me having to go through all that. (Maternity manager, health service 9)

We also talked about increasing midwifery skills, increasing our scope of practice, and because of that, we actually had a midwifery skills session in February, and [maternity educator name] came over, and we did, like application of Fetal Scalp Electrodes etc., so just to build up people’s skills. (Midwifery educator, health service 7)

Expanding on the Likert-type scaled statement responses that MANE had a positive impact on teamwork, several participants believed their health service already had good teamwork and collaboration within their unit, and that MANE helped to support this.

I feel that … regardless of their level of experience, our … even our midwifery students and junior midwives will escalate things if they’re worried about a situation, so I think that has certainly assisted with that process, yes. (Midwifery educator, health service 12)

#### The importance of maintaining MANE

Overall, MANE was viewed as an important program that should be maintained. This theme emerged when participants compared the MANE program to PROMPT, discussed mandating participation of services in MANE, and reflected on the barriers to running MANE.

##### Comparing MANE and PROMPT

Given that all but one of the health services included in the interviews were running PROMPT routinely during the MANE evaluation period, we explored participants’ views on both programs due to their similarities. While both are interdisciplinary simulation based obstetric emergency training programs, PROMPT is run within the health service, whilst MANE is run by external facilitators and also purports to have a stronger focus around clinical governance. Most participants agreed that there was a place in their health service for both MANE and PROMPT.

… there are certainly similarities, but their differences are what makes them both important. (Maternity manager, health service 13)

The use of PROMPT to support the learnings of MANE was also discussed by a number of interviewees.

I think you could say that [confidence increased] in the initial period [after MANE], but as we have a low number of births, I think that, that wanes off … if we were relying on MANE to achieve that as an ongoing thing I would say no, but … in combination with PROMPT … it reinforces it. (Executive, health service 6)

Some benefits of PROMPT over MANE were identified. The flexibility of the PROMPT program was discussed; being a shorter workshop (generally either half or one day programs) meant that it was easier to get staff to attend, and it could be scheduled when it suited the needs of the health service. Conversely, participants identified benefits that MANE had over PROMPT. MANE was viewed as a ‘more polished’, higher quality workshop than PROMPT and was seen to have better equipment and resources, superior expertise from educators, and higher quality, up-to-date presentations.

I think the video replay of the [scenario] cos (sic.) in PROMPT generally the spectators are generally in the room, where in MANE they’re outside the room, so yeah, not worrying about what people are saying or doing, and it’s not as crowded in the birth suite rooms as well … so more realistic I think you could say. (Maternity manager, health service 9)

As described above, the benefit of having external facilitators deliver MANE was raised repeatedly. MANE was viewed has having better ‘buy-in’ from attendees than PROMPT because the program was delivered by expert external facilitators.

We work with these people [in PROMPT] and we’re the ones sort of assessing them and telling them what to do and, so I definitely feel the outsiders coming in, they can point things out more clearly. (Maternity manager, health service 5)

Without any disrespect to the midwives running PROMPT, there’s that level of expertise and education experience in the MANE team that is just superior. (Maternity manager, health service 4)

Participants commented that PROMPT coordinators benefited from MANE as they could take these learnings and apply them to their PROMPT scenarios. MANE was also seen to be less work for the educators, who would also normally be responsible for organising PROMPT sessions.

##### To mandate or not?

Participants were asked their views on the mandate by the Victorian Department of Health for MANE to be run at all maternity services in rural and regional Victoria who provide care for women with low to medium obstetric risk. All but one participant could see benefit of mandating MANE, with one participant also reporting that they believed that the Department of Health had a responsibility for oversight of education across the maternity sector.

I think [MANE should be mandated] … seeing how much you can get out of it … the skills that clinicians can walk away with and feel confident about and learn from. (Maternity manager, health service 1)

The external facilitation of MANE was seen as one of the benefits of mandating the program.

Having somebody external come in and say this is what’s happening somewhere else rather than just running your PROMPT days where you’re really just looking internally at what you think you need to work on I think is really important for rural and regional hospitals that MANE is coming to. (Maternity manager, health service 9)

Of the 12 participants that discussed the frequency of MANE in the interview, five believed it should be mandated to be run annually, six bi-annually, and one every three years. Again, the need for both MANE and PROMPT programs was discussed, with suggestions that they be reviewed and restructured to improve the synergy between the two programs.

##### Barriers to running MANE

To establish the viability of MANE in the future, we asked interview participants to identify if there were any barriers to running MANE at their service. The cost of MANE, particularly in smaller health services, was a theme frequently identified as a barrier throughout the interviews.

Initially it was put off by the management because they didn’t want to pay the money when we can do PROMPT for a lot cheaper … but then … we were told that we had to run MANE at least every second year or something, so then they got them, and then now they’ve said we can book them again next year … that it’s worth the money. (Maternity manager, health service 5)

The operational impact MANE had on the health service was also identified by many participants as a barrier. This was particularly reflected in smaller health services where issues of staffing and rostering, and the capability of the maternity unit to operate during the two days the workshop was running were of concern.

We only have … ten midwives and when you think someone’s worked nightshift that night and someone [has to] work nightshift the next night and if someone’s in labour that’s three midwives who can’t attend. So, that cuts down who can attend. (Maternity manager, health service 10)

The difficulty in getting medical staff to attend the workshop was also reflected by several participants.

Our main [barrier to running MANE] is getting our medical staff attending … because they’ve all got other work elsewhere and commitments they have to keep and so on. (Maternity manager, health service 1)

### Response to Likert-type statements

The responses to five Likert-type statements supported the themes that were generated from the interview data, specifically that MANE *improved clinical practice* and *improved awareness and understanding of clinical governance*. In the three interviews where there was more than one participant, the participants all agreed on the rating to these statements. Participants agreed that MANE improved clinician’s skills (94%) and confidence (94%) in managing maternity and neonatal emergencies, and resulted to changes in clinical practice (70%). Additionally, participants agreed that MANE improved teamwork (82%), and improved clinicians’ confidence to escalate clinical concerns (94%).

## Discussion

This study described maternity managers’ and educators’ views of the MANE program. Overall, MANE was viewed positively by participants, and they considered that it resulted in positive changes in their health service. Both the Likert-type scaled statements and the open-ended responses suggest that MANE had a positive impact on clinicians, and more broadly on the way the maternity services operated.

One of the key reasons why participants in this study responded positively to MANE was the external facilitation, as they valued the expertise brought by MSEP and PIPER and saw benefit in having these external facilitators deliver education that was tailored to their service. Although the body of evidence on clinicians’ views of external facilitation is limited, one qualitative study exploring the roles of external facilitators and interprofessional facilitation teams on implementing evidence-based practice found that external facilitators were seen to provide support and constructive feedback and helped staff to overcome obstacles to build structures and processes in their service [21]. Although this study was in a different context in that the setting was not obstetric, the findings echoed participants’ responses in this study.

Managers and educators also expressed the view that the program had improved awareness and understanding of clinical governance by staff. Improving clinician’s understanding of clinical governance was one of the key aims of the MANE program. This aim originated from and was supported by the Victorian Clinical Governance Framework (VCGF). The VCGF was produced in 2017 as part the reforms introduced in response to the cluster of perinatal deaths discussed in the introduction to this paper [9]. The VCGF outlines that to achieve safe, high-quality care, all health service staff must engage in clinical governance at their health service [9]. The finding of this theme supports MANE in being an important factor in Safer Care Victoria’s long-term goal of improving the safety and care of Victoria’s rural and regional maternity services.

Participants in this study also reported that MANE led to improved clinical practice. These findings are consistent with the findings of the survey component of the evaluation, in which clinicians reported both immediate and sustained improvements in confidence and knowledge of managing emergencies post-MANE [16]. This maintenance of confidence and skills could be attributable in part to other factors that were identified by participants in the interviews, such as maintaining other education program throughout the year, i.e., PROMPT. The view of participants in this study that MANE had improved clinical practice is consistent with evaluations of other obstetric emergency education programs. In a systematic review assessing the effectiveness of training in emergency obstetric care, obstetric emergency training led to increased knowledge and skills and improved clinical practice [1]. The five clinical governance domains of the VCGF are leadership and culture; consumer partnerships; workforce; risk management and clinical practice [9]. The improvements to clinical practice support MANE as being an important factor to help health services address all of these domains.

Kumar et al.’s 2018 evaluation of PROMPT reported that participants of PROMPT had a positive learning experience and an increase in confidence in managing emergency obstetric situations from the PROMPT program [2]. Whilst most participants in this study agreed there was a place for both MANE and PROMPT, important differences between the two were established and justified the maintenance of both programs. While PROMPT was perceived as more flexible than MANE, several benefits of MANE compared with PROMPT were identified, including program quality, expertise of facilitators, access to different equipment and resources and the external oversight. The benefit of external oversight in highlighting the strengths of and areas of improvement for their health service also aligns with the VCGF domains of risk management and clinical practice [9]. The main barriers identified in running MANE impacted smaller services the most, with cost and staffing of the maternity unit during the program being challenging. The new maternity education program that has been implemented by MSEP to replace MANE, the Maternity Emergencies (ME) program, has taken this into consideration, and now runs the program over one day, with a focus on maternity emergencies only, and at a lower cost to the health service [22].

### Limitations

While this study overall found that maternity managers and educators viewed MANE positively, there are some limitations. The study was conducted with a small sample of maternity services that care for women and babies identified as being low to medium obstetric risk in rural and regional Victoria. The findings may not be generalisable to different settings including higher risk rural and regional health services, and metropolitan health services.

There are a number of factors that make it difficult to measure the influence of MANE alone on the outcomes described. There were different levels of engagement with PROMPT at each maternity service, making it difficult to ascertain whether changes were due to PROMPT or MANE, or both. Further, several services had introduced or were introducing other quality improvement projects in addition to MANE. It is therefore likely that improvements seen were due to a number of factors, with MANE being one.

The interviews varied in length, with a range of 12 to 36 min, possibly reflecting a difference in the level of engagement in the interview by some participants.

As described, it was important to determine the views of maternity managers and educators due to their influence on safety culture, clinical governance and program facilitation. This paper has only explored their views, and therefore other voices, such as clinicians and health service executives who have also been involved in MANE have not been reported in this paper.

### Implications for practice

This study has shown that maternity managers’ and educators’ views of MANE were positive and led to improvements in clinician’s awareness and understanding of clinical governance as well as improvements in clinical practice. MANE was seen as in important program to support the ongoing education of clinicians in health services. The views of the participants that support MANE in addressing the VCGF domains to improve patient safety should be considered when planning future maternity education programs across the state of Victoria and more broadly.

## Conclusions

The findings of this study highlight the benefits of an external education program such as MANE for smaller health services in regional and rural areas. MANE was viewed by maternity managers and educators very positively as an important program delivered by experts in their field. The participants viewed that the benefit of MANE over other education programs (such as PROMPT which is run within the health services) was the improvement in awareness of clinical governance, and the value of ‘external eyes’. Given the influence managers and educators have on the safety culture of their health service, the findings of this study are important in terms of programs such as MANE being delivered effectively, being sustained and ultimately making a positive influence on maternal and infant safety. Maternity managers and educators reported that clinicians’ skills and confidence improved as a result of participation in MANE. These findings from this primarily qualitative study that included 21 participants from 17 health services are consistent with those reported by maternity care clinicians (as reported by Cullinane et al. [16]).

## Data Availability

The datasets generated and/or analysed during the current study are not publicly available due to them containing information that could compromise research participant privacy and consent but are available from the corresponding author upon reasonable request.

## Abbreviations

MANE: Maternity and Newborn Emergencies
MSEP: Maternity Services Education Program
PIPER: Paediatric Infant Perinatal Emergency Retrieval
PROMPT: PRactical Obstetric Multi–Professional Training
SAQ: Safety Attitudes Questionnaire
SCV: Safer Care Victoria
VCGF: Victorian Clinical Governance Framework
